# Molecular dynamics simulations for mechanical properties of borophene: parameterization of valence force field model and Stillinger-Weber potential

**DOI:** 10.1038/srep45516

**Published:** 2017-03-28

**Authors:** Yu-Ping Zhou, Jin-Wu Jiang

**Affiliations:** 1 Shanghai Institute of Applied Mathematics and Mechanics, Shanghai Key Laboratory of Mechanics in Energy Engineering, Shanghai University, Shanghai, 200072, People’s Republic of China

## Abstract

While most existing theoretical studies on the borophene are based on first-principles calculations, the present work presents molecular dynamics simulations for the lattice dynamical and mechanical properties in borophene. The obtained mechanical quantities are in good agreement with previous first-principles calculations. The key ingredients for these molecular dynamics simulations are the two efficient empirical potentials developed in the present work for the interaction of borophene with low-energy triangular structure. The first one is the valence force field model, which is developed with the assistance of the phonon dispersion of borophene. The valence force field model is a linear potential, so it is rather efficient for the calculation of linear quantities in borophene. The second one is the Stillinger-Weber potential, whose parameters are derived based on the valence force field model. The Stillinger-Weber potential is applicable in molecular dynamics simulations of nonlinear physical or mechanical quantities in borophene.

Boron can be formed into a number of finite clusters due to plenty of chemical bonding for this element^1^. Some of these planar boron clusters were proposed as potential basis for the formation of single-layer boron (i.e. borophene)^2^,^3^,^4^. The structure of borophene on different substrates was predicted theoretically^5^,^6^, and was produced in recent experiments^7^,^8^,^9^,^10^.

Since then, intensive theoretical studies have been performed for various properties of the borophene. The stabilized structures of the borophene were investigated by first-principles calculations^11^,^12^,^13^,^14^,^15^,^16^,^17^. The interaction between borophene and various types of substrates plays an important role for the stability and physical properties of the borophene^18^,^19^,^20^,^21^,^22^,^23^,^24^,^25^. The electronic, optical and thermodynamic properties were investigated for the borophene^26^. It was demonstrated that there is no Schottky barrier between the metallic borophene and other two-dimensional semiconductors, which is useful for the construction of two-dimensional electronic devices with enhanced performance^27^. Borophene has negative Poisson’s ratio in the out-of-plane direction and the in-plane directions resulting from its puckered configuration^7^,^28^,^29^,^30^.

Besides the pure borophene, few approaches have been proposed to modify the electronic or phonon properties in borophene. For instance, hydrogenation can effectively tune the electronic current or other mechanical properties for the borophene^31^,^32^,^33^. Free edges in the borophene nanoribbon were found to be important for mechanical, electronic, magnetic, and thermal transport properties^34^,^35^,^36^. The strain effect has been studied for mechanical properties^28^,^37^,^38^, or magnetic properties in borophene^39^. Several defects were predicted to cause considerable effects on the anisotropic mechanical properties of the borophene^30^.

Along with the study of fundamental physical properties for borophene, there have also been some investigations on possible applications of borophene in the applied research fields. For example, the application of borophene as high capacity electrodes or anode materials was examined by several recent works^40^,^41^. First-principles calculations predicted possible superconductivity phenomenon in borophene due to the phonon-electron interaction^42^,^43^,^44^, which can be further manipulated by strain and carrier-doping^45^.

From the above literature survey, we find that most existing theoretical works are based on the first-principles calculations. These calculations are accurate, but are limited to small (or bulk) structures due to high computation requirements. As an alternative approach, the molecular dynamics simulation can be utilized to investigate very large systems typically containing more than 10^4^ atoms. The key ingredient in the molecular dynamics simulation is the interatomic interaction. The only one interaction potential available for the borophene is the ReaxFF force field model^46^. The present work aims to develop efficient linear and nonlinear empirical potentials for the borophene, which can assist further theoretical investigations for borophene of large size.

In this paper, we provide the valence force field (VFF) model and the Stillinger-Weber (SW) potential for the description of the interaction in borophene with low-energy triangular structure. The VFF model is a linear potential, which can be used to calculate elastic quantities such as the phonon dispersion. The SW potential is a nonlinear potential, which is derived based on the VFF model. The SW potential can be applied in the molecular dynamics simulation of nonlinear physical or mechanical properties for borophene. We demonstrate the usage of the SW potential with the publicly available LAMMPS package.

## Results

**Figure 1:**
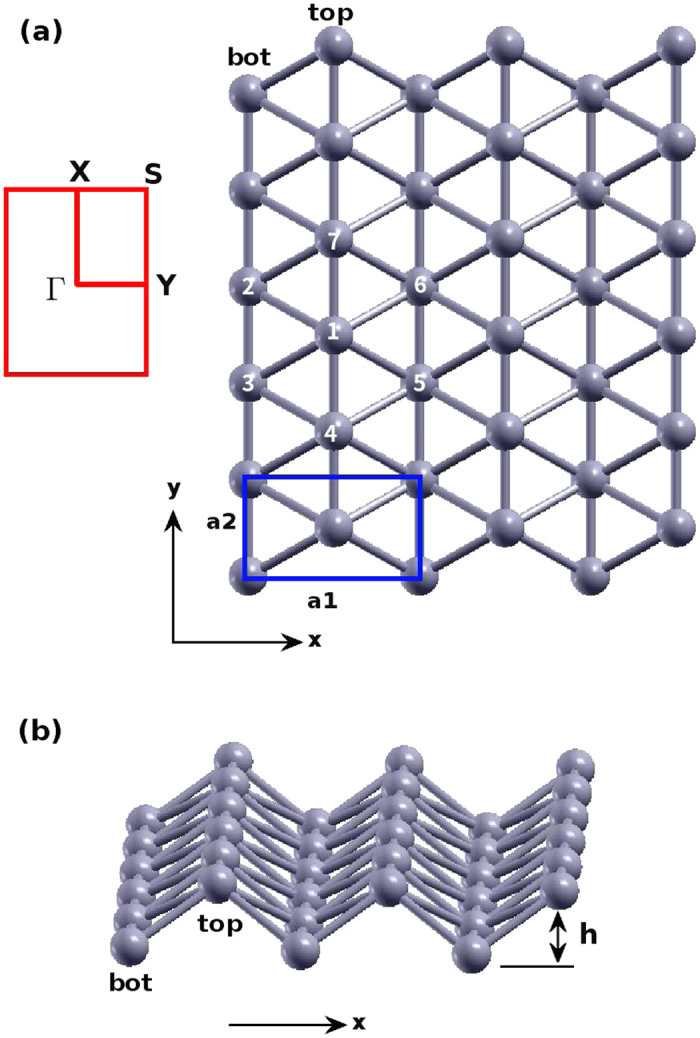
Structure for borophene. (**a**) Top view. Atoms are categorized into top chains and bottom chains. The top chain includes atoms like 1, 4, and 7. The bottom chain includes atoms like 2, 3, 5, and 6. The unit cell is shown by blue rectangle. The first Brillouin zone is shown by red rectangle on the left. (**b**) Perspective view illustrates the puckered configuration, with *h* as the distance between the top and bottom chains along the out-of-plane z-direction. The pucker is perpendicular to the x-axis and is parallel with the y-axis.

Figure 1 shows the structure of borophene, in which structural parameters are from the *ab initio* calculation^28^. Figure 1(a) shows the top view of the borophene with the rectangular unit cell in the xy plane. The two lattice bases are *a*_1_ = 2.866 Å and *a*_2_ = 1.614 Å. The first Brillouin zone is shown by red rectangle on the left. The two basic vectors for the reciprocal lattice are *b*_1_ = 2.192 Å^−1^ and *b*_1_ = 3.893 Å^−1^. There are two inequivalent boron atoms in the unit cell. Boron atoms are categorized into the top chain and the bottom chain. The top chain includes atoms like 1, 4, and 7. The bottom chain includes atoms like 2, 3, 5, and 6. Figure 1(b) discloses the puckered configuration of the borophene. The pucker is perpendicular to the x-direction, while it is parallel with the y-direction. The height of the pucker is *h* = 0.911 Å, which is the distance between the top chain and the bottom chain along the out-of-plane z-direction.

### Valence force field model

The VFF model is a useful linear model for the description of interatomic interactions in covalent materials, in which interactions are decomposed into some characteristic bond stretching and angle bending components^47^. These interaction components are in close relation with the vibration morphology of phonon modes. It is thus a proper approach to determine the VFF model for a covalent material based on its phonon dispersion. There are two typical terms for the VFF model, i.e., the bond stretching *V*_*r*_ and the angle bending *V*_*θ*_,


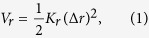



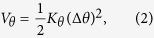


where Δ*r* and Δ*θ* are the small variations for the bond length and the angle. The two force constant parameters are *K*_*r*_ and *K*_*θ*_.

Table 1 shows five VFF terms for the borophene, four of which are the bond stretching interactions shown by Eq. (1) while the other two terms are the angle bending interactions shown by Eq. (2). The VFF model for the borophene is determined with the assistance of the phonon dispersion. The phonon dispersion for the borophene has been obtained by previous *ab initio* calculations. Parameters for the VFF model are determined by fitting the phonon dispersion to the *ab initio* calculations^28^. GULP^48^ is used for the computation of the phonon dispersion and the fitting of parameters in the VFF model.

**Figure 2:**
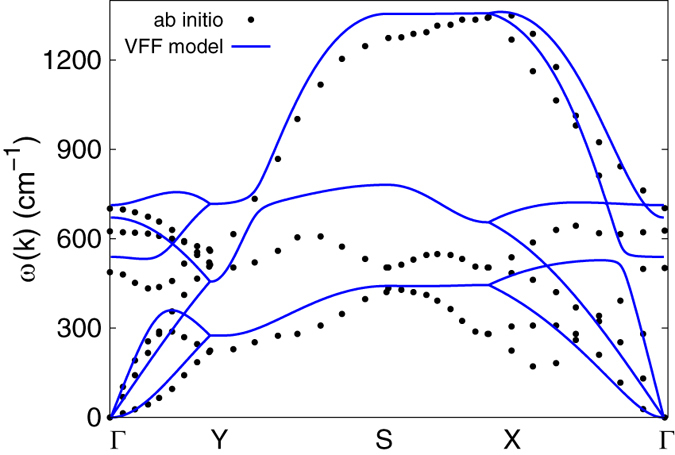
Phonon dispersion for borophene along ΓYSXΓ from the VFF model (blue lines) is compared to the data from the *ab initio* calculation (black dots)^28^. Phonon branches are doubly degenerate along YX due to the inverse symmetry between the top and bottom boron chains.

**Figure 3:**
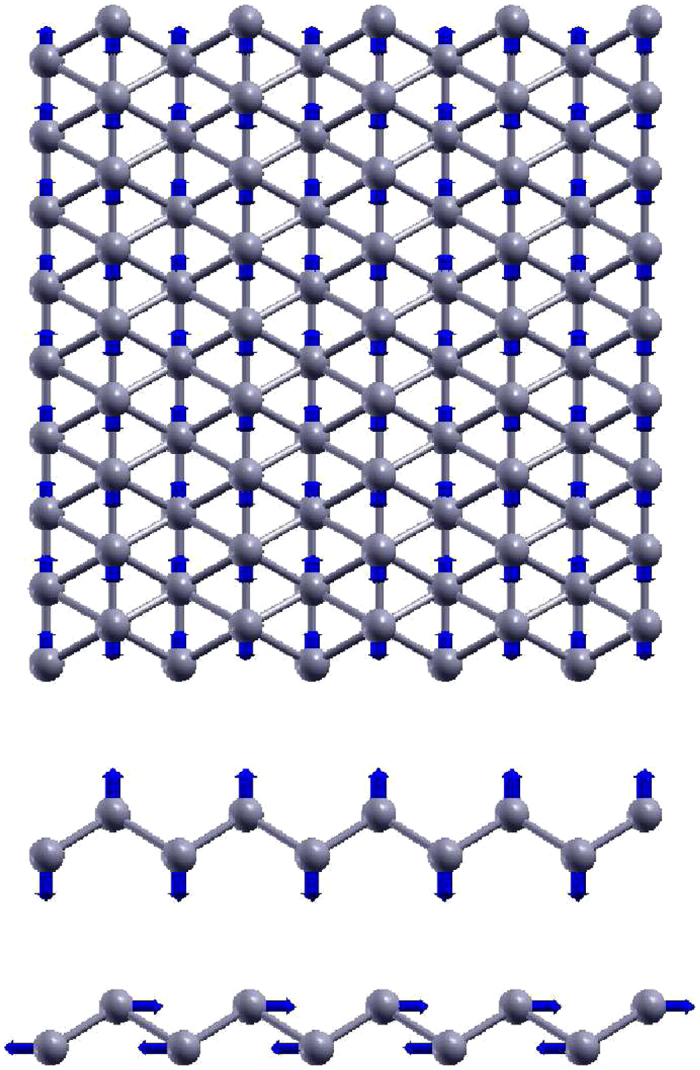
Three optical phonons at Γ point. From top to bottom, the frequencies are 539.2, 632.3, and 702.4 cm^−1^, respectively. Each atom chain vibrates as a rigid chain in these phonons. Arrow attached to the atom represents the vibration component of this atom.

**Figure 4:**
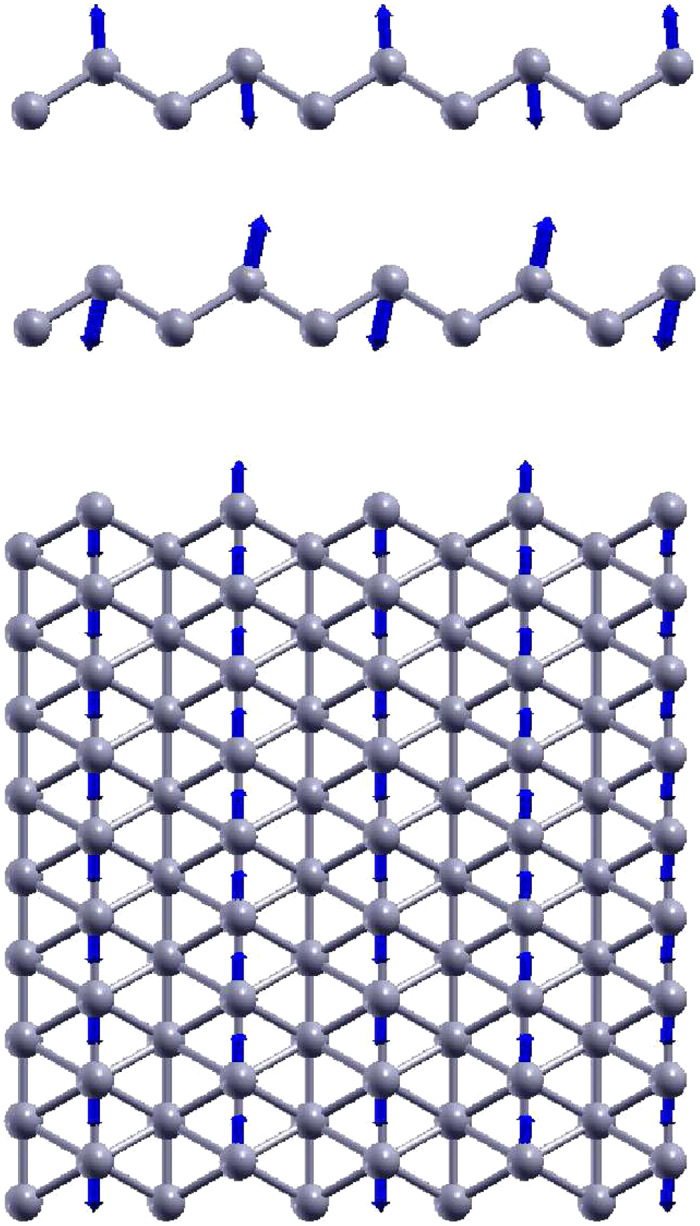
Three optical phonons at Y point. From top to bottom, the frequencies are 246.5, 386.1, and 589.4 cm^−1^, respectively. Each atom chain vibrates as a rigid chain in these phonons. The other three phonons at Y point have similar vibration morphology, which involves only atoms from the bottom chains.

Figure 2 shows that the phonon dispersion calculated from the VFF model is comparable with *ab initio* results. Good agreements are achieved for the three acoustic branches in the long wavelength limit. Similar phonon dispersion can be found from other *ab initio* calculations^26^,^29^,^38^. There is no imaginary mode from the VFF model, which appears in the *ab initio* calculations for the flexure mode in the long wave limit^26^,^28^,^29^,^38^. This imaginary mode is due to the long-range interaction in the borophene, which is not considered by the VFF model. Hence, there is no imaginary mode here. Figure 3 shows the vibration morphology of the three optical phonons at the Γ point. Each boron atom chain vibrates as a rigid chain in these phonons. The three phonon modes at the Y point are doubly degenerate, due to the inverse reflection symmetry between the top and bottom boron chains in the borophene. We have presented the vibration morphology for the three phonons at the Y point in Fig. 4, where only atoms from the top chains are involved in the vibration. For the other three phonons (not displayed here), only atoms from the bottom chains are involved in the vibration. Actually, the phonon branches are doubly degenerate along the whole YX line, due to the inverse reflection symmetry in the borophene.

**Figure 5:**
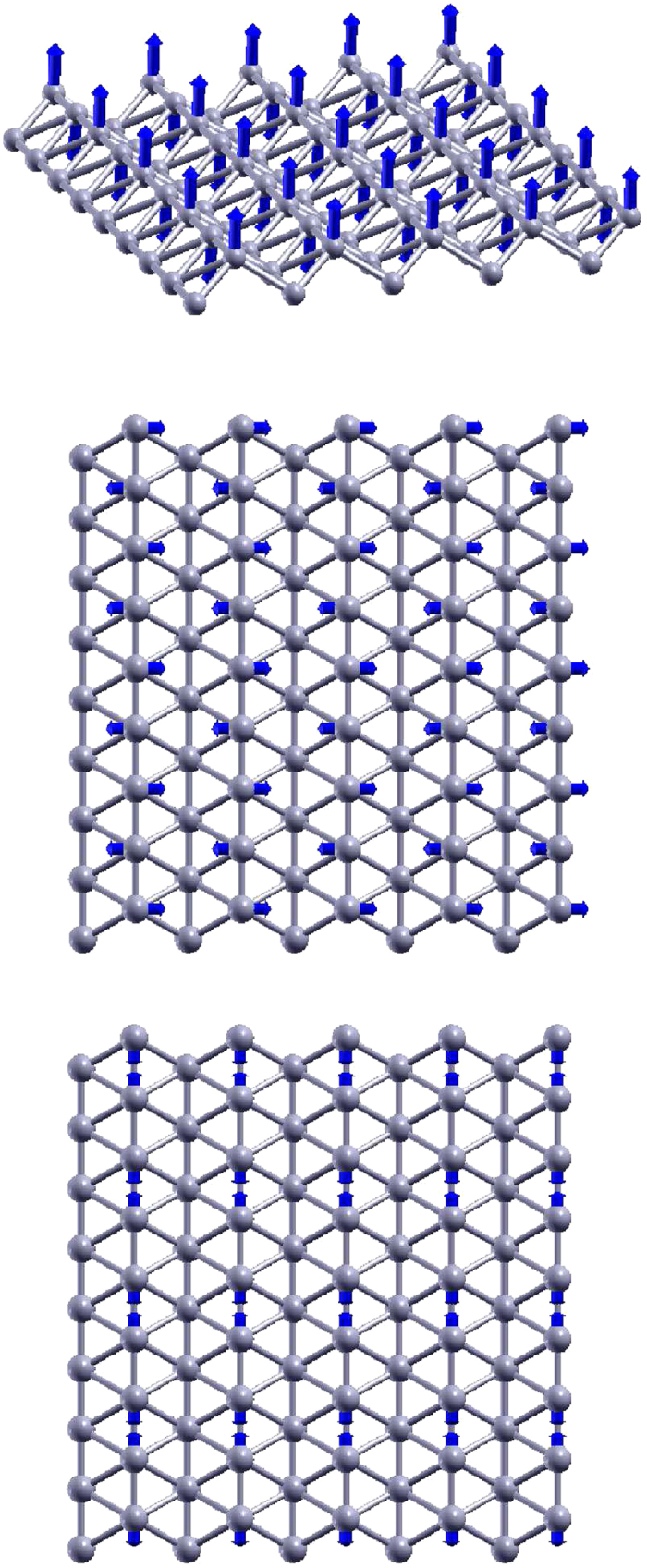
Three optical phonons at X point, involving vibration of atoms from the top chains. From top to bottom, the frequencies are 431.2, 603.5, and 1323.9 cm^−1^, respectively. The frequency of the intra-chain longitudinal optical phonon (1323.9 cm^−1^) is obviously higher than the two intra-chain transverse phonons. The other three phonons at X point have similar vibration morphology, which involves only atoms from the bottom chains.

An obvious feature in the phonon dispersion of the borophene shown in Fig. 2 is that the highest-frequency branch (around 1300 cm^−1^) along SX has much higher frequency than the other branches. Figure 5 shows the vibration morphology of these phonons at the X point. The highest-frequency phonon is the intra-chain optical vibration; i.e., neighboring boron atoms within the same chain are vibrating in an out-of-phase manner. It means that the intra-chain bonds like *r*_14_ in Fig. 1(a) are much stronger than the inter-chain bonds like *r*_12_. This anisotropy is reflected in the force constant parameters for the VFF model listed in Table 1. The intra-chain parameter *K*_14_ is larger than the inter-chain parameter *K*_12_ by a factor of four, so the resultant frequency for the intra-chain optical phonons (around 1300 cm^−1^) is about twice of the frequency for the inter-chain optical phonons (around 700 cm^−1^) shown in Fig. 2.

### Parameterization of the SW potential

The VFF model presented in the previous section describes the linear component of the interaction within borophene, but it does not provide any information for the nonlinear interaction. The SW potential is one of the most efficient nonlinear potentials. The SW potential includes both linear and nonlinear interactions. One of the present authors (J.W.J.) has shown that the linear component of the SW potential can be derived analytically based on the VFF model^49^. A distinct feature of this analytic approach is that the parameters of the SW potential are governed by a constraint, which guarantees that all bonds and angles are fully relaxed in the initial configuration. The constraint is shown as follows,


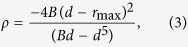


where *d* is the equilibrium bond length, and other quantities are potential parameters. The nonlinear component of the SW potential is determined by the parameter *B*, which can be determined by the third-order nonlinear constant.

**Figure 6:**
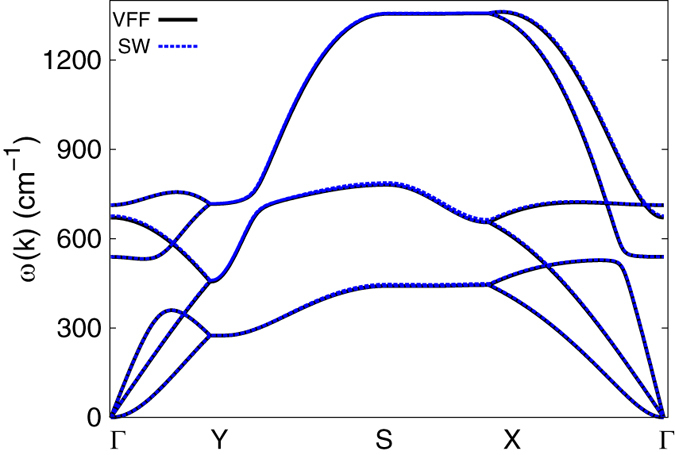
Phonon dispersion from the VFF model and the SW potential.

The parameters for the two-body SW potential can be derived from the bond stretching terms in the VFF model. The obtained parameters are shown in Table 2. The parameters for the three-body SW potential are obtained from the angle bending terms in the VFF model. The parameters are shown in Table 3. Using this SW potential, we calculate the phonon dispersion for the borophene, which is the same as the phonon dispersion calculated using the VFF model as shown in Fig. 6. It indicates that the SW potential has inherited the linear interaction of the VFF model.

**Figure 7:**
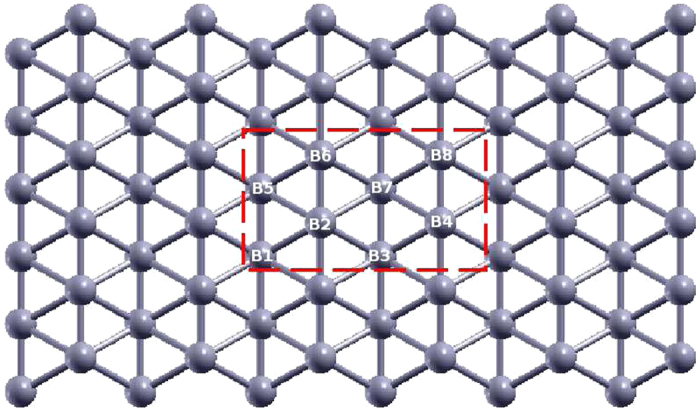
Eight atom types are introduced for the boron atoms in the borophene. Atoms B_1_, B_3_, B_5_, and B_7_ are from the bottom chain. Atoms B_2_, B_4_, B_6_, and B_8_ are from the top chain.

The publicly available package LAMMPS is widely used for MD simulations. The SW potential has a slightly different form in LAMMPS. The parameters for the SW potential can be determined by comparing the potential forms in GULP and LAMMPS. Table 4 shows some representative parameters for the SW potential used by LAMMPS. It should be noted that we have introduced eight atom types for boron atoms in the borophene as shown in Fig. 7. It is because the cut off (4.0 Å) for the two-body SW potential between atoms like 3–5 is larger than the distance between atoms 3–6 (3.289 Å). However, there is no interaction between atoms like 3–6. It is thus necessary to differentiate bonds like 3–5 and 3–6 with atoms 3 and 6 denoted by different atom types, so that the two-body SW potential is not imposed on the bonds like 3–6. There are 512 lines (categorized into six classes) in the SW potential script used by LAMMPS. We have shown in Table 4 one representative term for each of these six classes. These files for LAMMPS can be found in the Supplemental materials, including the SW potential script (borophene.sw), an input file for LAMMPS (in.tension), and a structure generation code (xyz.f90).

**Figure 8:**
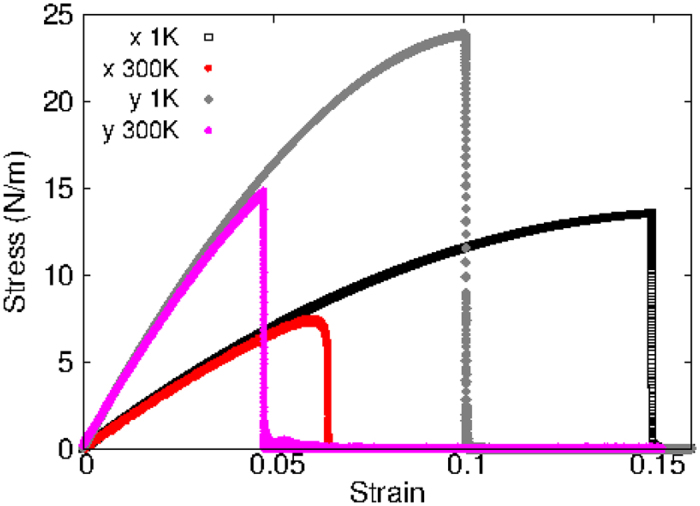
Stress-strain relations for the borophene of size 100 × 100 Å. The borophene is uniaxially stretched along the x or y directions at temperatures 1 K and 300 K.

**Figure 9:**
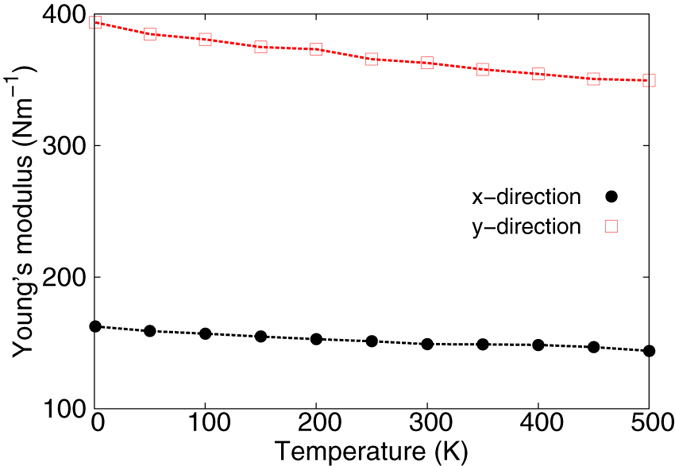
The temperature dependence for the Young’s modulus in the borophene along the armchair and zigzag directions.

Figure 8 shows the stress strain relations for the borophene of size 100 × 100 Å. The structure is uniaxially stretched in the x or y directions at 1 K and 300 K. The Young’s modulus is 163 Nm^−1^ and 394 Nm^−1^ in the x and y directions respectively at 1 K, which are obtained by linear fitting of the stress strain relations in [0, 0.01]. These values are in good agreement with the *ab initio* results at 0 K temperature, eg. 170 Nm^−1^ and 398 Nm^−1^ in ref. 7, or 166 Nm^−1^ and 389 Nm^−1^ in ref. 28, or 163 Nm^−1^ and 399 Nm^−1^ in ref. 50. Figure 9 shows that the Young’s modulus decreases with the increase of temperature. Previous *ab initio* calculations obtained negative Poisson’s ratio for the uniaxial stretching of the borophene in the x and y directions, eg. −0.02 and −0.04 in refs 7,28. The Poisson’s ratio from the present SW potential are −0.011 and −0.029, which are quite comparable with the *ab initio* results.

The yielding strain is about 0.15 and 0.1 in the x and y directions respectively at the low temperature of 1 K. These values agree with *ab initio* results at 0 K^28^,^38^,^50^. The yielding strain decreases with increasing temperature. The nonlinear parameter *B* = 0.6*d*^4^, with *d* as the bond length, is determined by the third-order nonlinear constant (*D*) along the x-direction. The third-order nonlinear constant is obtained by fitting the stress strain relation to the function 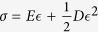, with *E* as the Young’s modulus. The obtained value of *D* is −944 Nm^−1^ along the x-direction, which is fitted to the *ab initio* result of ref. 28 −924 Nm^−1^. The obtained value of *D* is −2785 Nm^−1^ in the y direction.

**Figure 10:**
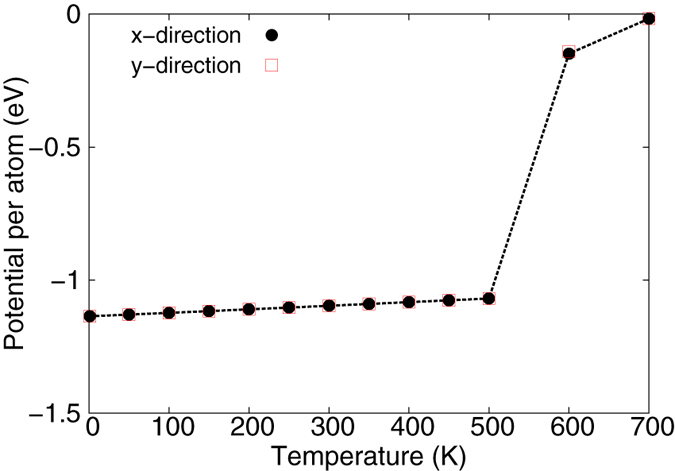
Potential energy per atom in borophene at different temperatures. A sudden jump at the critical temperature *T*_*C*_ = 550 ± 50 indicates the instability of the borophene above *T*_*C*_.

**Figure 11:**
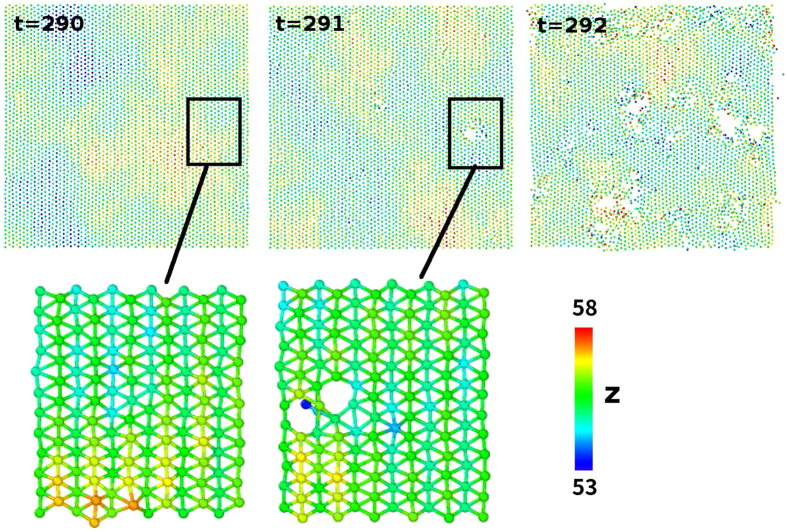
The destruction process for the borophene at 600 K. The colorbar is with respective to the atomic z-coordinate.

The SW potential is not able to describe accurately the complicate chemical bonding processes. However, it contains reasonably accurate nonlinear components, so it is able to provide some qualitative descriptions for the thermal-induced nonlinear phenomena. As an example, Fig. 10 shows the total potential energy in the borophene at varying temperatures. A sudden jump at the critical temperature *T*_*C*_ = 550 ± 50 K reveals that the borophene is stable at temperatures bellow the critical temperature *T*_*C*_. However, the structure becomes unstable for temperatures above the critical temperature. Figure 11 shows the destruction process for the borophene at 600 K. Some boron atoms have obviously larger thermal vibration amplitudes at *t* = 290 ps. These boron atoms will be evaporated at *t* = 291 ps, which results in a vacancy defect in the borophene. The whole borophene is destroyed when there are too many vacancy defects at *t* = 292 ps.

We note that the VFF model and the SW potential parameterized in the present work are only applicable to describe the interaction for the triangular phase of the borophene, but can not be applied to other phases for the borophene.

## Discussion

To summarize, we have developed two empirical potentials to describe the interaction between boron atoms within borophene. The first one is the linear VFF model, which is determined based on the phonon dispersion of the borophene. The VFF model is suitable for the calculation of linear quantities in borophene. Starting from the VFF model, we derive the second empirical potential, the SW potential, to describe the interaction for borophene. The SW potential includes both linear and nonlinear couplings, so it is applicable for the simulation of some nonlinear quantities. We also provide some necessary files for the application of the SW potential by using LAMMPS.

## Method

### One minor improvement for the implementation of three-body SW potential in LAMMPS

We discuss a practical improvement for the three-body SW potential implemented in LAMMPS. Let’s take angles *θ*_143_ and *θ*_137_ in Fig. 1 as an explicit example to demonstrate this improvement.

It is obvious that these two angles should be quite different. As shown in Table 3, there is a three-body SW potential for *θ*_143_, but there is no interaction for *θ*_137_. The values of these two angles are quite different in the equilibrium configuration; i.e., 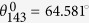 and 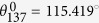. However, their arm lengths are the same; i.e., *r*_13_ = *r*_13_ and *r*_14_ = *r*_17_. In the three-body SW potential implemented in LAMMPS, angles are distinguished by their two arm lengths, so angles *θ*_143_ and *θ*_137_ become indistinguishable. Consequently, the three-body SW potential (intent solely) for *θ*_143_ will also be applied to *θ*_137_, which causes a technical issue.

In general, it will become important to distinguish two angles constructed by the same type of atoms, when there are more than two different types angles around each atom. Similar situation also occurs in the simulation of MoS_2_ using the three-body SW potential implemented in LAMMPS. We have proposed to distinguish each angle by its opposite arm length^49^,^51^. That is, the bonds *r*_34_ and *r*_37_ are quite different in length, so they can be used to distinguish angles *θ*_143_ and *θ*_137_. However, this bond length based criterion is an ad hoc technique, as the bond length is a structural dependent parameter. We note that this criterion is also adopted by GULP^48^.

Here, we provide a more universal criterion to distinguish two angles. It is based on the input initial value *θ*_0_ for the angle of the three-body SW potential. For example, in Table 3, the input initial value for the three-body SW potential regarding *θ*_143_ is 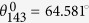. For a given angle *θ*, if the following condition is satisfied, then the three-body SW potential will be applied to this angle,


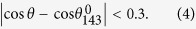


It can be checked that *θ*_137_ does not obey the criterion in Eq. (4) and it is thus excluded from the three-body SW potential. The angle *θ*_143_ obeys the criterion in Eq. (4) under moderate deformations. Hence, the three-body SW potential is applied to the angle *θ*_143_, while this three-body SW potential is not applied to the other angle *θ*_137_.

The criterion in Eq. (4) can be implemented into LAMMPS by modifying its pair_sw.cpp source file in the following two steps. A continuous cut-off function in the range of [0.25, 0.35] has been introduced to avoid possible boundary effects in molecular dynamics simulations.


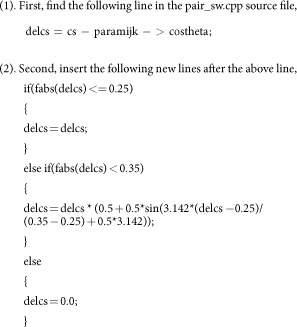


Then recompile the LAMMPS package. The recompiled LAMMPS executable file can be used to simulate borophene with the SW potential parameterized in the present work. It can also be used to simulate MoS_2_ correctly using its SW potential. We note that this criterion does not affect other normal simulations using LAMMPS, where it is not necessary to distinguish angles. We expect this criterion to be implemented in LAMMPS in its future versions.

## Additional Information

**How to cite this article:** Zhou, Y.-P. and Jiang, J.-W. Molecular dynamics simulations for mechanical properties of borophene: parameterization of valence force field model and Stillinger-Weber potential. *Sci. Rep.* **7**, 45516; doi: 10.1038/srep45516 (2017).

**Publisher's note:** Springer Nature remains neutral with regard to jurisdictional claims in published maps and institutional affiliations.

## Supplementary Material

Supplementary Dataset

## Figures and Tables

**Table 1:** The VFF model for borophene.

VFF type	Bond stretching				Angle bending	
expression	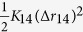	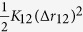	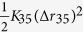	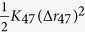	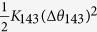	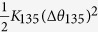
parameter	15.873	4.025	1.828	1.411	2.523	1.150
*r*_0_ or *θ*_0_	1.614	1.880	2.866	3.228	64.581	99.318

The second line gives an explicit expression for each VFF term, where atom indexes are from Fig. 1 (a). The third line is the force constant parameters. Parameters are in the unit of 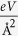 for the bond stretching interactions, and in the unit of eV for the angle bending interaction. The fourth line gives the initial bond length (in unit of *Å*) for the bond stretching interaction and the initial angle (in unit of degrees) for the angle bending interaction. The angle *θ*_*ijk*_ has atom i as the apex.

**Table 2:** Two-body SW potential parameters used by GULP^48^.

	*A* (eV)	*ρ* (Å)	*B* (Å^4^)	*r*_min_(Å)	*r*_max_ (Å)
*r*_14_	5.135	0.769	4.207	0.0	2.050
*r*_12_	1.402	0.618	6.246	0.0	2.419
*r*_35_	2.957	2.065	36.096	0.0	4.000
*r*_47_	1.683	1.413	65.146	0.0	4.100

The expression is 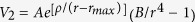. The quantity (*r*_*ij*_) in the first line lists one representative term for the two-body SW potential between atoms i and j. Atom indexes are from Fig. 1(a).

**Table 3:** Three-body SW potential parameters used by GULP^48^.

	*K* (eV)	*θ*_0_ (degree)	*ρ*1 (Å)	*ρ*2 (Å)	*r*_min12_ (Å)	*r*_max12_ (Å)	*r*_min13_ (Å)	*r*_max13_ (Å)	*r*_min23_ (Å)	*r*_max23_ (Å)
*θ*_143_	28.382	64.581	0.769	0.618	0.0	2.050	0.0	2.419	0.0	2.419
*θ*_135_	5.852	99.318	0.618	0.618	0.0	2.419	0.0	2.419	2.240	3.047

The expression is 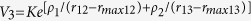 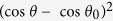.

The first line (*θ*_*ijk*_) presents one representative term for the three-body SW potential. The angle *θ*_*ijk*_ has the atom i as the apex. Atom indexes are from Fig. 1(a).

**Table 4:** SW potential parameters used by LAMMPS^52^.

	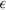 (eV)	*σ* (Å)	*a*	*λ*	*γ*	cos*θ*_0_	*A*	*BL*	*p*	*q*	tol
B_1_-B_5_-B_5_	1.000	0.769	2.666	0.000	1.000	0.000	5.135	12.030	4	0	0.0
B_1_-B_2_-B_2_	1.000	0.618	3.914	0.000	1.000	0.000	1.402	42.820	4	0	0.0
B_1_-B_3_-B_3_	1.000	2.065	1.937	0.000	1.000	0.000	2.957	1.985	4	0	0.0
B_1_-B_1_-B_1_	1.000	1.413	2.902	0.000	1.000	0.000	1.683	16.343	4	0	0.0
B_1_-B_8_-B_5_	1.000	0.000	0.000	28.382	0.000	0.429	0.000	0.000	0	0	0.0
B_1_-B_8_-B_6_	1.000	0.000	0.000	5.852	0.000	−0.162	0.000	0.000	0	0	0.0

The two-body potential expression is 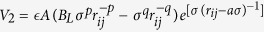. The three-body potential expression is 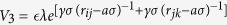

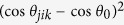
. Atom types in the first column are displayed in Fig. 7.
